# Transforming the Indian Private Sector for Universal Health Coverage

**DOI:** 10.3390/healthcare13212802

**Published:** 2025-11-04

**Authors:** Nachiket Mor

**Affiliations:** Banyan Academy of Leadership in Mental Health, Chennai 600037, India; nachiket@nachiketmor.net

**Keywords:** health–system integration, managed competition, contingency-based strategy, resource-based view, dynamic capabilities, institutional theory, hospital-first model, primary-care-first model, emerging health systems

## Abstract

Background/Objectives: India’s private healthcare sector remains fragmented, with weak primary care, uneven secondary services, and tertiary care accessible to few. Fee-for-service payments and indemnity-style insurance distort prices and fragment accountability. This paper develops a conceptual, theory-driven framework for integrating financing and delivery so that prices reflect social opportunity costs and competition rewards value rather than volume. Methods: A comparative synthesis of international integration models covering Israel, the United States, Spain, Brazil, and the United Kingdom was undertaken. Each exemplar was analysed for ownership form, market maturity, and regulatory capacity, and interpreted using four strategic management theories: Contingency theory, the Resource-based view, Dynamic capabilities, and Institutional theory. These perspectives were combined to construct a contingency-based typology tailored to India’s mixed health system. Results: Two state-contingent integration pathways emerged. Hospital-first vertical integration suits hospital-dense, high-growth states such as Tamil Nadu and Delhi, where capital and regulatory depth permit managed-care scaling. Primary-care-first reverse integration is preferable in resource-constrained contexts such as Bihar and Chhattisgarh, leveraging community trust and lower capital intensity. Conclusions: Achieving universal health coverage in India requires regulatory conditions, such as ownership flexibility, solvency oversight, risk adjustment, and transparent outcomes reporting, to enable accountable payer–provider organisations to form. The framework extends contingency theory to mixed health systems and offers a transferable blueprint for emerging markets seeking sustainable, integrated managed care.

## 1. Introduction

### 1.1. Current Status of the Indian Healthcare System

As discussed in the 2019 NITI Aayog report [1], “Building Blocks of a Health System for New India” [1], India’s health system rests primarily on two pillars: the public and private sectors (including non-profits and cooperatives). The public sector has overtones of the Russian Semashko model of the 1920s, characterised by features such as detailed, line-by-line budget-based financing and salaried providers [2,3]. It maintains an active presence across all relevant health system spheres, from One Health to quaternary care [4,5], and is reasonably well funded [6]. However, it requires multiple reforms to be implemented before it can function to its full potential. The private sector is characterised by fragmentation and incoherence. Primary care (first-contact, community-based, and preventive services that manage most common health needs and coordinate referrals) is plentiful [7,8]. It is, however, delivered mainly through solo practitioners and informal providers, with minimal continuity, quality assurance, or gatekeeping [9,10]. Secondary care (specialist outpatient and inpatient services for conditions requiring short-term or general hospital management, including emergency care) is available in urban and peri-urban areas. However, it is often of medium quality and poorly coordinated. Tertiary care (high-specialty, technology-intensive services such as advanced surgeries, oncology, and critical care) is of higher quality but is located in large urban areas and, due to very low insurance penetration, is not accessible to most of the population [11].

Given the range of services provided by the public sector, the most feasible strategy for India would be to expand and completely reorganise it, just as many low- and middle-income countries (LMICs) in Asia, Africa, and Latin America have done, so that it can offer Universal Health Coverage (UHC) to the entire population both as a financier and a provider. This effort needs to be prioritised. The private sector will also need to play a role in India’s UHC journey as a provider of healthcare services. In this paper, the focus is on private healthcare that is privately financed, with the implicit assumption that, in the Indian context, public financing is exclusively reserved for the public healthcare sector. The “perfect-market” [12] structure of the private healthcare sector in India, characterised by myriad providers, unrestricted consumer choice, and the predominance of a fee-for-service payment mechanism, has, however, led to poor health outcomes and low levels of financial protection. This is essentially because healthcare suffers from high information asymmetries and distorted consumer preferences [13], making it difficult for free markets to allocate resources and organise themselves in a manner that results in the best possible healthcare outcomes.

### 1.2. The Rationale for Integrated Managed Care

The policy question, therefore, is how to structure the private sector so that prices offer meaningful signals. Integrated managed care is an approach that can move Indian health markets closer to the conditions under which prices approximate social opportunity costs [13,14], i.e., come closer to becoming a “sufficient statistic” [15]. This approach to organising healthcare in a market-based environment strengthens price-based competition. It consolidates fragmented, distortion-prone prices into a single plan premium that internalises prevention and coordination benefits. Within a plan, primary and hospital budgets are unified, so investments in community care that avert admissions lower the plan’s own costs. With risk adjustment and open enrolment, the observed premium more closely reflects social opportunity cost. Information asymmetry is mitigated because purchasers compare plan-level outcomes and access indicators, rather than relying on opaque per-service quality. This reduces the principal violations that block the First Welfare Theorem (the proposition that price-based competition in any market results in the most efficient outcomes) in healthcare. These include externalities, market incompleteness, and asymmetric information [13]. As a result, competition across integrated plans has a better chance of delivering efficient allocations than competition across fragmented providers (see Table A1 in the Appendix A for a listing of the principal academic arguments). The experience of Israel’s health funds [16,17,18,19] and the Netherlands’ post-2006 regime [20] illustrates how regulated, private, managed competition can make the premium a near-sufficient statistic for performance, provided risk selection is constrained and outcomes reporting is credible [14,21]. Interestingly, since integration provides predictable, annuity-type revenue streams (capitation, bundled premiums) compared with episodic fee-for-service, it also has the potential to attract greater investor interest [22]. However, despite its apparent attractiveness to both consumers and investors, Indian private markets have not yet moved in this direction. This is principally because hospitals and insurers are locked into fee-for-service models [1]. Additionally, highly risk-averse, low-ambition incumbent investors, providers, and insurers harbour the mistaken belief that they benefit from the currently high levels of fragmentation [23]. Without an integration strategy that links financing with the organisation of care, India risks remaining in a fragmented equilibrium.

### 1.3. International Models of Integration

Internationally, two contrasting models of integration can be observed. The first is hospital-first vertical integration, where hospitals expand downstream into primary care and insurance. Examples include Geisinger in the United States, Clalit in Israel, and the Alzira partnership in Spain. The second, less frequently theorised, is primary-care-first reverse integration, in which physician groups consolidate primary care and progressively contract for secondary and tertiary services; exemplars include physician-led Accountable Care Organisations (ACOs) in the United States, GP (General Practitioner) commissioning in the United Kingdom, CareMore’s risk-stratified model in the United States, and Maccabi in Israel.

There are clear benefits to hospital-first strategies, as demonstrated by the examples of Geisinger’s Patient-Centered Medical Home (PCMH) model [24,25,26,27]; the Presbyterian Hospital-at-Home model [28]; Clalit in Israel [29]; Alzira in Spain [30,31]; and multiple models in Brazil [32,33]. However, there is a concern that with hospital-first models, prices could rise, and quality gains may be uncertain, driven by steering patients to higher-priced facilities and shifts in facility-fee revenues [34,35,36,37,38,39,40,41].

Evidence from mature managed-care systems consistently indicates that primary-care-led integration produces greater cost savings than hospital-led models. In the United States, physician-group Accountable Care Organizations (ACOs) achieved larger and more durable Medicare savings than hospital-based ACOs [42,43,44,45]. In the United Kingdom, GP-fundholding and commissioning schemes lowered prescribing and referral expenditures relative to centrally managed or hospital-dominated arrangements [46,47,48]. Long-standing delegated-risk models in California likewise demonstrate sustained utilisation control and financial stability within capitated physician organisations [49,50,51]. Across these diverse contexts, primary-care-anchored entities have demonstrated stronger leverage over utilisation, chronic disease management, and downstream spending—supporting the proposition that reverse integration from primary care can yield superior cost efficiency when compared with hospital-first approaches. However, this has primarily been documented in the U.S./U.K. institutional contexts and has not been synthesised as a general strategy blueprint for emerging markets.

### 1.4. Potential Integration Pathways for Indian Providers

From the global experiences discussed above, Indian providers have two potential pathways to choose from:

(1) Hospital-First Integration: Here, regional or family-owned hospitals expand downstream into primary care and insurance. The advantages of this approach include leveraging the local hospital’s capital base and brand strength, which make it easier to obtain financing for such an endeavour, given the hospital’s existing financial streams.
(2) Primary-Care-First Reverse Integration: Strong, Starfield-consistent, i.e., comprising the “4Cs” of “coordination, first contact of care, continuity of care and comprehensive care” [52,53], primary networks built first, expanding upstream into secondary/tertiary contracting. The advantages of this approach include lower costs, preservation of trust, and better management of the burden of chronic diseases. The challenges are that such efforts are harder to initiate due to India’s weak primary care baseline and require robust financial support.

### 1.5. Research Question

To help policymakers and providers determine the most effective integration pathways, this paper examines a contingency-based conceptual framework for integration strategies in mixed health systems, with India serving as the focal case. The study aims to determine how India’s private sector, utilising these multiple pathways, can transition from fee-for-service fragmentation to integrated managed care through context-contingent strategies.

While Enthoven’s managed competition model and the WHO’s integrated, people-centred care framework both seek to link financing with service delivery, they approach integration at the macro-system level. Enthoven envisaged competition among health plans operating under a single national purchaser and uniform benefit rules [14,20]. The WHO framework [54] outlines normative principles, namely, coordination, continuity, comprehensiveness, and people-centredness, but does not specify how organisational ownership forms or market structures condition feasibility. This paper seeks to contribute to the existing literature by shifting the unit of analysis from system design to organisational strategy, formalising how hospitals and primary-care networks can pursue context-contingent pathways to integration within diverse state-level environments.

### 1.6. Organisation of the Paper

The following section outlines the theoretical foundations and explains how four strands of strategic management theory, namely, Contingency theory, the Resource-based view, Dynamic capabilities, and Institutional theory, jointly inform the framework. Subsequent sections apply this conceptual model to global exemplars and to state-level archetypes in India, followed by a discussion of policy implications and future research directions.

## 2. Conceptual Design and Analytical Approach

This study is conceptual and theory-building in design. Rather than testing hypotheses empirically, it develops a framework through comparative synthesis of global experiences with health-system integration. The analytical process involved the identification of international exemplars of hospital-first and primary-care-first integration through a targeted review of academic and policy literature, the abstraction of the structural and governance features of these models, and the interpretation of these features through the lens of strategic-management theory to derive a contingency-based typology applicable to India.

This analytical process constitutes a theory-driven comparative synthesis. It combines principles from conceptual typology construction and structured, focused comparison. Each exemplar was examined using a common set of guiding questions:

(1) What organisational mechanisms link financing and delivery?
(2) How is risk distributed across providers and payers?
(3) What contextual contingencies (ownership, market maturity, or regulation) shape viability?

Insights from this structured comparison were then abstracted into a typology linking contextual conditions to strategic responses. This stepwise, transparent procedure enhances replicability and analytical rigour, allowing future researchers to reproduce or extend the framework by applying the same criteria to additional cases. The quantitative indicators cited throughout the paper are illustrative, drawn from published case analyses to clarify mechanisms rather than to provide new empirical estimates.

The study draws on the following strands of strategic management theory to inform the selection of models for the Indian private sector within each of the above pathways.

(1) Contingency theory: Strategy must be tailored to the regional conditions of market growth, competition, and institutional capacity [55].
(2) Resource-based view (RBV): Strong primary care networks represent rare and difficult-to-imitate assets that can serve as foundations for reverse integration [56].
(3) Dynamic capabilities: Organisations must reconfigure capabilities as they extend upstream or downstream [57].
(4) Institutional theory: Legitimacy with regulators and funders is critical when providers seek to integrate financing [58].

Among these, Contingency theory provides the overarching logic of fit between organisational strategy and environmental context. RBV explains how distinctive internal assets, such as trust, data systems, or clinical reputation, enable organisations to execute integration more effectively. Dynamic capabilities clarify the process of reconfiguring these assets as providers extend upstream or downstream, while Institutional theory highlights the role of legitimacy and regulatory alignment in sustaining such transitions. This hierarchy of theories ensures conceptual coherence and avoids treating them as parallel or competing explanations.

The application of these theories draws on selected international exemplars chosen for their relevance to mixed or transitional health systems. Inclusion was based on three pragmatic criteria, namely, documented integration of financing and delivery under capitation or population-based payment, clear organisational data on ownership and governance, and peer-reviewed or authoritative policy documentation. Each exemplar, such as Clalit, Geisinger, Alzira, Unimed, CareMore, or Maccabi, illustrates a different configuration of integration rather than serving as a dataset for empirical analysis. This approach maintains conceptual rigour while grounding the framework in observed institutional diversity.

The choice of a conceptual rather than empirical design reflects the current evidence environment. Comparable, high-quality data on costs, clinical outcomes, and regulatory performance across India’s diverse private-sector providers are not yet available, and most international integration studies are case-based rather than cross-nationally standardised. A theory-driven, comparative-synthesis approach therefore provides a transparent way to organise the scattered evidence into a coherent analytical framework while clearly signalling the need for subsequent empirical validation. This choice ensures that the framework remains grounded in observed experience while avoiding premature quantification based on incomplete data.

This manuscript has made deliberate use of artificial intelligence (AI)-assisted tools (ChatGPT 5) to support the research process. AI was employed to synthesise large volumes of academic and policy literature, identify international case exemplars, and organise them into comparative evidence tables. However, care was taken to ensure that all references were independently verified against original sources and that the author critically evaluated all the arguments. AI was thus used as an augmentative tool for efficiency and breadth of coverage, not as a substitute for scholarly judgement.

## 3. Application of the Theoretical Models to Each Integration Pathway

Building on this theoretical foundation, the following subsections apply these logics to hospital-first and primary-care-first integration pathways.

### 3.1. Hospital-First Integration Pathway

The strategic question for regional hospitals considering managed care integration is fundamentally one of fit. In strategic management, Contingency theory argues that there is no universal “best way” to organise; rather, effectiveness depends on aligning organisational structures and governance with environmental contingencies [55,59]. Organisations that achieve this alignment demonstrate superior performance, while those that do not, experience instability and inefficiency. In the context of regional hospitals, two critical contingencies emerge: market growth potential and competitive intensity. Hospitals in low-growth, low-competition environments are unlikely to attract investor capital for aggressive expansion and may instead benefit from emphasising stewardship through non-profit or cooperative structures. By contrast, hospitals operating in high-growth, high-competition contexts must pursue capital-intensive strategies and align with investor expectations, making for-profit models more feasible. This leads to a contingency-based typology in which ownership form and strategic orientation are matched to market conditions.

However, contingency fit alone does not explain why some regional hospitals succeed in transitioning toward managed care while others fail. Here, insights from RBV and Institutional theory become critical. RBV emphasises that organisations achieve sustained competitive advantage not merely by aligning with external contingencies but by leveraging unique, valuable, and hard-to-imitate internal resources [56]. For regional hospitals, such resources include deep community trust, embedded local reputation, and long-standing physician relationships. These intangible assets are difficult for corporate chains to replicate. They may give regional hospitals a distinct advantage in launching primary–care–based managed care programmes, especially where patient loyalty and trust are decisive factors. However, RBV also highlights limitations: without access to financial capital or advanced IT systems, these hospitals may find it challenging to raise the necessary resources and develop the core capabilities required for scaling.

Institutional theory complements these perspectives by foregrounding the importance of legitimacy. Organisations survive not only by being efficient but also by conforming to societal and regulatory expectations [58,60,61]. In India and other emerging markets, community hospitals operating as non-profits or cooperatives often enjoy normative legitimacy as custodians of local health, whereas for-profit entrants must establish pragmatic legitimacy with investors and regulators. The choice of ownership form thus becomes not merely a financial decision but a question of how hospitals signal legitimacy to multiple audiences: patients, regulators, payers, and capital markets.

Together, these three theoretical lenses, i.e., Contingency theory, RBV, and Institutional theory, provide a structured foundation for analysing strategic options. Contingency theory specifies when different ownership forms are appropriate; RBV explains how some hospitals can execute managed care transitions by leveraging unique internal resources; and Institutional theory clarifies why governance choices resonate differently across contexts of trust, regulation, and public perception.

### 3.2. Primary-Care-First Integration Pathway

Most literature on healthcare integration in emerging markets examines hospital systems that expand downstream into primary care. This paper also explores an alternative strategic pathway, i.e., primary care providers who, by virtue of trust and longitudinal patient relationships, expand upstream into secondary and tertiary services through managed care integration. Drawing on RBV, Dynamic capabilities theory, and Institutional theory, this paper argues that primary care mastery constitutes a rare and hard-to-imitate capability that can anchor system-wide growth. The paper proposes a staged model whereby primary care organisations integrate with regional secondary hospitals, outsource tertiary care through reverse-bidding contracts, and replicate the model regionally. This framework highlights the strategic advantages of primary-care-first integration in delivering value, achieving legitimacy, and ensuring scalability in emerging markets such as India.

RBV emphasises that sustained competitive advantage arises from resources that are valuable, rare, hard-to-imitate, and non-substitutable [56]. In emerging markets, the scarce capability is not hospital infrastructure but rather trust-based, Starfield-consistent primary care, characterised by first-contact accessibility, comprehensiveness, continuity, and coordination [62]. Corporate hospital chains often fail in this regard, making it a defensible competitive advantage for primary care providers. While RBV highlights what resources matter, Dynamic capabilities theory explains how organisations reconfigure resources in changing environments [57]. Primary-care-first integration requires capabilities to sense opportunities for upstream partnerships and insurance contracting, and to seize them through new organisational arrangements and by transforming delivery models to maintain patient-centredness while scaling regionally. This dynamic reconfiguration is critical to scaling a regional proof-of-concept into a replicable growth model.

Institutional theory underscores the role of legitimacy [58,60,61]. In India, primary care–first providers can more easily secure normative legitimacy (as community stewards) than hospital-first entrants, whose expansion often attracts scepticism over commercial motives. Insurance integration and reverse-bidding for tertiary care contracts could also confer regulatory legitimacy.

## 4. Comparative Analysis of Integration Pathways

Appendix B provides a detailed discussion of the various global typologies of hospital-first models, while Table A2 presents examples of global hospital-first integrated care models and their performance outcomes. Table A3 presents an analysis of the evidence comparing primary-care-first and hospital-first models. Table A4 presents global examples of primary-care-first integrated models, with commentary on their potential relevance to the Indian context. Table A5 presents an analysis of the different dimensions of hospital-first and primary-care-first managed care pathways, along with a comparison between them.

### 4.1. For Hospital-Based Providers

Building on the theoretical foundations discussed above, this paper proposes a contingency-based framework that positions hospitals along two dimensions: market growth potential and competitive intensity, and derives four archetypal strategies. These archetypes are then empirically illustrated through comparative international cases and subsequently applied to the Indian context (Table 1). Applying this matrix to the Indian case suggests the strategies outlined in Table 2.

### 4.2. For Primary Care Providers

From the theoretical analyses, the following strategic pathway emerges for primary-care-first integration:

- Stage 1: Consolidation of Primary Care: Build Starfield-consistent models focusing on continuity, comprehensiveness, and data systems. Secure brand equity around trust and prevention.
- Stage 2: Selective Upstream Integration: Partner with or acquire regional secondary hospitals, while retaining patient-flow control in primary care. Introduce shared clinical protocols and referral systems.
- Stage 3: Tertiary Care Contracting: Develop insurance pools at the community or employer level. Use reverse-bidding contracts to purchase tertiary services from competing providers, ensuring cost efficiency and quality.
- Stage 4: Regional Replication: Export model to new regions, leveraging primary care competence and brand. Embed learning systems for continuous improvement.

This approach works with, among other things, patient trust as a key anchor, which is stronger than hospital-first models in fragmented markets; asset-light integration requiring lower capital expenditure compared to hospital expansion; cost control by leveraging insurers’ partnerships for tertiary contracting; and scalability, since primary care know-how travels better across regions than hospital infrastructure.

For India, this model resonates with policy shifts toward primary care [63]. Regional family-owned or cooperative providers may be uniquely positioned to pursue this pathway. The model offers a route to combine social legitimacy with financial sustainability, creating hybrids that serve both community and investor needs. Primary-care-first integration represents a theoretically grounded, practically feasible alternative to hospital-driven managed care in emerging markets. By leveraging rare resources (RBV), dynamically reconfiguring them (Dynamic capabilities), and aligning with institutional legitimacy requirements (Institutional theory), such providers can extend their value proposition while remaining asset-light. The pathway provides both a strategic contribution to management theory and a practical blueprint for strengthening India’s health system.

## 5. Discussion: Theoretical and Policy Interpretation

Despite the compelling policy and financial logic for integrated managed care, India’s private sector incumbents face structural barriers. Large tertiary systems heavily depend on doctors acting as consultant free agents, which limits their ability to align incentives around outcomes. Smaller family-owned hospitals are preoccupied with short-term survival under reimbursement ceilings, leaving little bandwidth for longer-term repositioning. Primary care providers are thinly capitalised and lack actuarial or contracting skills, making them hesitant to assume gatekeeper roles. Digital platforms remain transactional brokers rather than population health managers. These constraints explain why, despite all arrows pointing to integration, movement has been limited.

The Contingency theory-based approach proposed in this paper offers a way forward. In hospital-dense, high-growth states such as Tamil Nadu or Delhi, hospital-first integration is feasible if paired with strong guardrails to prevent cost inflation and monopolistic behaviour. In states such as Chhattisgarh or Bihar, where hospital density is low and trust deficits are high, a primary-care-first approach is more suitable, leveraging community legitimacy and lower capital requirements. The financial and operating models chosen by them will also need to vary, with non-profit and cooperative models perhaps being more suited to low-growth and stable markets, while a for-profit/PE (Private Equity)-backed approach may work better for dynamic, competitive urban markets, where the rewards may be higher but so are the risks.

These implementation patterns are not merely contextual but reinforce the contingency logic of the framework. In regions with dense hospital networks, implementation challenges revolve around aligning multiple capital-intensive entities under common governance, whereas in hospital-scarce settings, the primary challenge is institutionalising trust and accountability in nascent physician-led networks. Thus, regional variation illustrates the underlying theoretical proposition that structural context determines the viable integration pathway.

For both pathways, regulation is the binding constraint. Without ownership flexibility, solvency frameworks for delegated risk, risk adjustment, and transparent quality reporting, integration will not materialise. Policymakers must therefore move beyond expanding indemnity-style insurance and instead create enabling conditions for accountable, integrated organisations to emerge. Furthermore, while regulation is a necessary precondition, political and institutional realities also have a role in determining whether integration succeeds. In states with strong medical lobbies or fragmented regulatory oversight, vested interests may resist risk transfer and quality transparency. Provider organisations accustomed to fee-for-service revenue face high transition costs, while insurers may fear the loss of control associated with delegated risk. These dynamics underscore that integration is not merely a technical reform but a political–economic process shaped by bargaining among regulators, providers, and investors.

Trust and responsiveness to community needs are central to the success of integrated models. Evidence from ACOs and GP commissioning indicates that primary-care-led models accumulate trust through continuity, personal relationships, and responsiveness to local preferences. In contrast, hospital-led integration may risk community trust when financial integration is perceived to prioritise revenue over patient welfare. In India, where patient loyalty is often personal rather than institutional, the ability to preserve and scale relational trust may be decisive in determining which integration pathway achieves legitimacy. Responsiveness, understood as alignment with community health priorities, becomes both a precondition and a product of successful integration. Quality, in this sense, extends beyond clinical metrics to include perceived fairness, continuity, and emotional safety—dimensions often neglected in conventional efficiency analyses.

Implementation feasibility will also vary by state. Tamil Nadu and Delhi can experiment with hospital-first integration because their regulatory and capital ecosystems are relatively mature. In contrast, states such as Chhattisgarh and Bihar will need phased, primary-care-first approaches built on cooperative or trust-based governance. Recognising these state-contingent pathways transforms the framework from a one-size-fits-all blueprint into a realistic strategy portfolio. The examples in Table A6 in the Appendix B illustrate both the barriers and the opportunities for Indian incumbents in a potential shift toward managed competition.

Beyond regulatory design, financial sustainability is a central determinant of long-term viability. From a financial perspective, integrated managed care can stabilise provider revenues and support long-term investment in preventive and chronic care. Traditional fee-for-service hospitals generate volatile, transaction-based income, whereas capitation and membership models produce predictable, annuity-like cashflows that enhance solvency and allow for counter-cyclical investment in community care. Such cashflows command higher valuation multiples and support longer debt tenors, making them attractive for both private equity and long-term institutional capital [22]. From an investor’s perspective, integrated managed care is therefore not only a policy imperative but also a financial opportunity.

## 6. Conclusions and Future Research

This paper examines how India’s fragmented private health sector might evolve toward universal health coverage through two distinct but complementary integration pathways—hospital-first and primary-care-first. Using Contingency theory as the overarching frame, and drawing on insights from the Resource-based view, Dynamic capabilities, and Institutional theory, it developed a typological framework that links ownership form, hospital density, market growth, and regulatory capacity to strategic choice. The central proposition is that no single model fits all Indian states or all types of providers: feasible strategies depend on contextual alignment between organisational capabilities and institutional environments.

### 6.1. Theoretical and Policy Contributions

The framework advances theory by extending Contingency theory to the domain of mixed health systems and illustrates how internal resources and external legitimacy jointly determine integration success. For policy, it offers a diagnostic tool for regulators and providers to assess state-level readiness. Rather than prescribing a uniform template, it identifies conditional pathways that align financial sustainability with equitable access—providing a structured alternative to ad hoc insurance expansion. In contrast to Enthoven’s system-wide model of managed competition [14,20] and the WHO’s framework on people-centred integrated care [54], which focus on national design principles, this paper locates integration within providers’ strategic choices. It extends managed-competition theory by identifying the contextual variables, such as hospital density, regulatory capacity, ownership form, and market growth, that determine which integration model is viable in mixed health systems. This meso-level focus bridges macro-policy frameworks and the micro-foundations of organisational behaviour.

India’s health system stands at a crossroads. The coexistence of weak primary care, uneven secondary care, and shallow insurance penetration has locked the country into a fragmented equilibrium. While recent reforms have expanded coverage, they have not articulated a coherent strategy for integration. This paper has argued that two distinct pathways are available to the Indian private sector: hospital-first vertical integration and primary-care-first reverse integration.

The originality of this contribution lies in three areas. First, it formalises both pathways as strategy options for LMICs, a context in which even hospital-first integration is still in its early stages. Second, it embeds these models within a contingency framework that links market growth, hospital density, ownership form, and regulatory capacity to strategy choice. Third, it positions India as a focal case for emerging-market managed care innovation, highlighting that the absence of exemplars from developing countries itself constitutes a gap in the literature.

From this analysis flow several propositions: hospital-first integration can succeed in dense, high-growth markets such as Tamil Nadu if accompanied by strong regulatory guardrails; primary-care-first integration may deliver greater value in resource-constrained settings such as Chhattisgarh where continuity and trust are paramount; ownership form conditions viability, with non-profit and cooperative structures suited to stable markets and for-profit governance more appropriate in growth contexts; and regulators who create enabling conditions through risk adjustment, solvency oversight, and transparent quality reporting, can expand the feasible set of strategies.

For India, the implication is clear: universal health coverage cannot be achieved solely by expanding insurance coverage. Progress depends on fostering integrated, accountable organisations capable of managing care across the life course. By offering a contingency-based framework that is both conceptual and practical, this paper provides a blueprint not only for India but also for other emerging health systems facing the same challenge of transitioning from fragmented provision to sustainable managed care.

### 6.2. Implications for Regulators and Government

For integrated payer–provider organisations to take root in India, the regulatory environment will need to undergo fundamental changes. Currently, rules are designed for large, capital-intensive insurers and fragmented fee-for-service hospitals, leaving little room for regional hospitals or physician groups to experiment with managed care. A first step is to allow insurers to own and operate hospitals, and hospitals to own insurers, so that incentives for population health are aligned rather than fragmented. Alongside this, minimum capital requirements should be reduced to enable smaller and cooperative entities, including not-for-profits, to participate in risk-bearing arrangements [64]. Individual risk-based underwriting should remain prohibited, but insurers should be permitted to underwrite geographically and design narrow networks that focus on coordination and quality.

Such changes would need to be coupled with more sophisticated oversight. A credible risk adjustment system is essential to ensure that insurers compete on efficiency rather than enrolment strategies. At the same time, delegated risk frameworks, similar to California’s solvency rules for physician groups, are necessary if hospitals or clinics are to take capitated payments. Regulators must also mandate transparent quality reporting so that patients and purchasers can compare outcomes, and competition authorities should actively monitor mergers and network restrictions to prevent monopolistic behaviour. Beyond ownership and solvency rules, space must be created for cooperative and trust-based health plans, which have proven effective in countries such as Brazil but are currently excluded in India.

Together, these measures would create an enabling environment for hospitals, physician groups, and insurers to evolve into genuine managed-care organisations, capable of delivering both financial protection and continuity of care.

### 6.3. Future Research Directions

This conceptual framework generates several questions that future empirical studies could address:

(1) Effectiveness of hospital-first strategies: Under what combinations of hospital density, market growth, and regulation do hospital-anchored models reduce costs without inflating prices?
(2) Performance of primary-care-first models: Can physician-led or cooperative networks achieve superior chronic disease outcomes and financial protection in hospital-scarce states?
(3) Ownership and legitimacy: How do non-profit, cooperative, and for-profit forms differ in their ability to gain regulatory and community trust?
(4) Regulatory levers: Which combinations of risk adjustment, solvency oversight, and quality reporting expand the feasible set of integration strategies?
(5) Comparative testing: Cross-country studies could validate whether this contingency typology generalises to other emerging markets.
(6) Mixed-method approaches: Policy experiments, state-level comparative analyses, and simulation modelling could be used to test the conceptual propositions advanced in this work empirically.

## 7. Limitations

This paper is conceptual and therefore carries several limitations:

(1) While the comparative framework of hospital-first and primary-care-first integration pathways is grounded in international evidence, it has not been empirically tested in the Indian context. The propositions advanced here should be interpreted as hypotheses that require validation through future mixed-methods research, including case studies, pilot evaluations, and econometric analysis of regional variations.
(2) The evidence base itself is uneven: much of the global literature focuses on high-income settings such as the United States, Israel, and Western Europe, while emerging market cases are fewer and less rigorously evaluated. Extrapolating from these examples to India necessarily involves assumptions that may not fully capture India’s unique institutional and political-economy realities.
(3) The financial modelling of potential pathways is indicative rather than definitive, as reliable data on private sector costs, margins, and capital flows in India remain scarce.
(4) Although the framework foregrounds regulatory and policy requirements, it does not fully engage with the political challenges of reform implementation, such as entrenched interests among tertiary specialists, hospital associations, and state-level actors. These limitations underscore the need for further empirical research, stakeholder engagement, and policy experimentation to test and refine the propositions offered in this paper.

## Figures and Tables

**Table 1:** Archetypal strategies for hospital-first integration (Global).

	Low Competition	High Competition
Low growth	Non-profit stewardship (mission-driven, stable annuity revenues, community legitimacy). Clalit (Israel); Geisinger (U.S. rural Pennsylvania).	Niche alliances (affiliations with larger networks to defend share; focus on differentiation). Marshfield Clinic (U.S., Wisconsin, rural oligopoly)
High growth	Hybrid models (non-profit hospital with for-profit insurance arm; leverage capital but protect trust). Unimed (Brazil cooperatives); SSM/Dean Health (U.S. Midwest).	For-profit expansion (investor-backed scaling, replication across regions). Hapvida NotreDame Intermédica (Brazil); Rede D’Or–SulAmérica.

**Table 2:** Archetypal strategies for hospital-first integration (India).

	Low Competition	High Competition
Low growth	Non-profit stewardship (mission-driven, stable annuity revenues, community legitimacy). For tier-2 regional markets with limited growth, hospitals should emphasise non-profit stewardship, building managed care for stability and legitimacy.	Niche alliances (affiliations with larger networks to defend share; focus on differentiation). Maybe the best strategy for single specialty hospitals.
High growth	Hybrid models (non-profit hospital with for-profit insurance arm; leverage capital but protect trust). In Indian Peri-urban growth corridors, hybrid models (hospital trust + for-profit insurer subsidiary) may balance capital needs with community trust.	For-profit expansion (investor-backed scaling, replication across regions). Corporate chains (Apollo, Fortis, Narayana) dominate metros and may need to integrate with owned insurance programmes.

## Data Availability

All data used in this research are available in the study.

## Appendix A

**Table A1:** How Integrated Managed Care helps private healthcare markets function better.

Arrow–Debreu Condition ^a^	Fragmented Market	Integrated Managed Care	Effect on Price Sufficiency
No externalities	Prevention benefits not priced into hospital budgets	Unified budget internalises spillovers	Improves
Complete markets	Incomplete insurance; gaps in coverage	Insurance bundled with provision	Improves
Symmetric information	Patients observe little; providers game claims	Plan-level outcomes more observable	Improves
Price-taking Behaviour	Concentrated hospitals; bilateral market power	Competing plans disciplined by switching (if regulated)	Mixed: depends on entry/antitrust
Convex technologies	Induced demand under fee-for-service	Global budgets, care pathways	Improves

^a^: Arrow–Debreu conditions for the optimal functioning of markets are drawn from the foundational paper by Arrow and Debreu [65].

## Appendix B. Typologies of Global Hospital-First Models

### Appendix B.1. Hospital-Anchored Integrated Delivery Networks (IDNs) with Owned Plans (U.S.)

Regional systems that run both hospitals and a licensed health plan. These include Geisinger with its transformed primary care model [24], Presbyterian with its ‘Hospital at Home’ model [28]; Marshfield [66], HealthPartners [67,68], and Dean Care [69,70].

(1) Geisinger (Pennsylvania). Geisinger’s ProvenHealth Navigator (PHN) embeds nurses/care managers in PCMHs, with analytics-driven risk management, supported by Geisinger Health Plan. Peer-reviewed studies report reduced costs and improved clinical quality under PHN [24,26], while system-level analyses describe the broader innovation and EHR foundation.
(2) Presbyterian Healthcare Services (New Mexico). A nine-hospital system with a substantial health plan; its “Hospital-at-Home” and complex-care programmes illustrate primary-care-anchored management of high-risk patients. A Health Affairs study reported 19% lower costs with equal/better outcomes for Hospital-at-Home vs. inpatient comparators in the Presbyterian model [28,71].
(3) Marshfield Clinic Health System (Wisconsin). Physician-hospital system operating Security Health Plan; strategic documents explicitly align community health priorities with SHP plan operations—an example of a rural/regional scale IDN [66].
(4) HealthPartners (Minnesota/Wisconsin). Consumer-governed integrated payer–provider that runs Regions Hospital and HealthPartners insurance; official materials document plan/provider integration and payer operations [67,68].
(5) SSM Health/Dean Health Plan (Wisconsin). Regional IDN historically aligned with Dean Health Plan (now administered by Medica); organisational materials emphasise population health and integrated care management [69,70].

The U.S. cases show that owning the health plan enables empanelment, PCMH-based risk management, and utilisation control inside care, such as Geisinger’s PHN and Presbyterian’s Hospital-at-Home, producing measurable cost/quality gains [24,28].

### Appendix B.2. Verticalized Payer–Provider Groups (Brazil)

In this model, the insurer owns/operates hospitals, clinics, and diagnostics under one platform. These include Hapvida [72,73] and the non-profit cooperative UNIMED [74].

(1) Hapvida–NotreDame Intermédica (HNDI). Brazil’s largest private health platform, after its merger. Regulatory filings and ratings reports describe it as a verticalized model combining plans with owned hospitals/clinics/diagnostics, citing competitive cost advantages from scale and integration [72,73].
(2) Rede D’Or + SulAmérica. Brazil’s largest hospital chain acquired major insurer SulAmérica; CADE cleared the deal in December 2022. This move consolidated hospital–insurer integration at a national scale and catalysed further consolidation in diagnostics and insurance [72].
(3) Unimed (regional cooperatives). City/state physician-owned cooperatives operate both plans and provider networks (hospitals/clinics). Unimed-BH (Belo Horizonte) alone covers ~1.28 million clients with an extensive provider network—an existence proof of regional payer–provider integration [33,74].

The Brazilian cases show that verticalization supports cost control and standardisation; Brazil’s regulator (ANS) and market structure have enabled insurers to internalise provision, with evidence of efficiency advantages and intense consolidation cycles.

### Appendix B.3. Capitated Regional Concessions (Spain)

A single entity provides primary and hospital care for a defined population under capitation—with Alzira in Valencia being the most well-known example from Spain [30,31]. The Alzira model integrated primary care and hospital services under per capita payment for a defined population (Valencia region). Research documents both the innovative coordination (single IT/records, unified pathways) and the political-economic factors that led to its reversion to public management in 2018–19. From the Spanish model, for regional hospitals, the key lesson is that the capitation-anchored, end-to-end accountability for population outcomes across care levels is feasible and can deliver enhanced value to the populations it serves [30,31].

### Appendix B.4. Nationwide HMO with Owned Hospitals (Israel)

Clalit integrates financing with system-wide primary care and hospitals. Clalit is a non-profit HMO that both finances and delivers care, operating nationwide primary-care clinics and owning hospitals. Israel’s Health System Review profiles Clalit’s model (and other HMOs) as payers-and-providers with strong IT and quality infrastructure—arguably the most mature end-to-end example globally [29].

### Appendix B.5. Cross-Cutting Operating Model (What “Good” Looks Like)

(1) Empanelled, primary-care-first design: PCMH teams with named clinician accountability, registries, proactive recall, and same-day access for risk-tiered panels. (US PCMH evidence base; Geisinger PHN) [24].
(2) Risk stratification & embedded care management: Attribution, predictive models, and nurse/care managers integrated into the clinic flow; Presbyterian’s Hospital-at-Home as an escalation pathway [28].
(3) Utilisation management inside care: E-consults, referral criteria, formulary alignment, and home-/virtual-first pathways governed by clinical leadership, as exemplified by Geisinger and Clalit [27,29].
(4) Verticals that matter: On-site diagnostics, pharmacy, and select specialty lines matched to the book of risk as demonstrated by the Brazilian verticalization experience [72,73].
(5) A financing arm (plan) to capture annuity revenue: Premiums fund upstream care; local/regional plans allow for contracting agility, such as in US IDNs and Brazilian merged entities [66,72]. Owning the premium stream stabilises cash flows, supports counter-cyclical investment in primary care, and can improve debt capacity; market observers explicitly link competitive advantage in Brazil to scale plus verticalized models (e.g., Hapvida). Hospital–insurer combinations (e.g., Rede D’Or/SulAmérica) reflect investor bets on synergy and margin capture along the value chain. While multiples vary by macro conditions, antitrust, and execution risk, the annuity-like nature of premiums is central to the equity story [72].

International precedents demonstrate that regional hospitals can operate capitated, end-to-end models that include owning the plan, strengthening primary care, embedding risk management, and coordinating secondary care, yielding cost/quality benefits, as well as annuity-like revenue. The design is portable and tailored to local regulations. Table A2 summarises the insights from global hospital-first models.

**Table A2:** Examples of hospital-first Integrated Care.

System	Own Plan?	Primary-Care Model	High-Risk Management	Evidence/Notes
Geisinger (US-PA)	Yes	PCMH/PHN	Embedded nurses; analytics	Cost ↓, quality ↑ in PHN [24].
Presbyterian (US-NM)	Yes	PCMH + home-first	Hospital-at-Home	19% cost ↓ vs. inpatient [28].
Marshfield (US-WI)	Yes (Security Health Plan)	Rural PCMH	Care coordination	IDN docs align with SHP [66].
HealthPartners (US-MN/WI)	Yes	Total-cost-of-care	Complex-needs clinics	Integrated payer/provider [67,68].
SSM/Dean (US-WI)	Yes (historically)	Pop-health programmes	Care management	Integrated IDN messaging [69,70].
Hapvida–NDI (BR)	Yes	Family-doctor lines	Verticalized ops	Vertical model documented [73].
Unimed-BH (BR)	Yes (co-op)	Primary-care gatekeeping	Regional network	1.28 M covered; integrated [33,74].
Alzira/Ribera (ES)	Capitated concession	Integrated PC + hospital	Risk tools; unified IT	PPP concession model [30,31].
Clalit (IL)	HMO (payer–provider)	Multidisciplinary clinics	Population Analytics	Own hospitals + clinics [29].

**Table A3:** Evidence matrix: hospital-first vs. primary-care-first integration.

Theme	Geography	Key Finding	Source	Method/Design	Strength/Notes
Physician-led ACOs vs. hospital-led ACOs	USA (Medicare)	Physician-group ACOs generated net savings to Medicare by year 3, which were larger than those of hospital-led ACOs.	[75]	Quasi-experimental analysis of MSSP ACO spending	Highly cited; rigorous methods; federal claims data
Physician-led ACOs vs. hospital-led ACOs	USA (Medicare)	Early years show substantially greater savings for physician-group ACOs.	[42]	Difference-in-differences evaluation	Found more substantial savings in physician-led models
Physician-led ACOs performance (synthesis)	USA (Medicare)	Independent physician-led ACOs associated with larger savings than hospital-led ACOs.	[43]	Evidence synthesis of ACO evaluations	Authoritative synthesis; policy relevance
Small practices in ACOs	USA (Medicare)	Small practices participating in ACOs controlled costs more than larger practices.	[45]	Observational analysis of ACO performance	Suggests primary-care-first feasibility at small scale
GP-led commissioning (CCGs)	UK (NHS)	GP involvement adds value to commissioning (referrals, prescribing); outcomes mixed, context dependent.	[76]	Realist evaluation (qualitative/quantitative mixed)	Mechanisms for GP ‘added value’ identified
GP-led commissioning (CCGs)	UK (NHS)	Explores how and when GPs add value to commissioning decisions.	[77]	Policy research report (realist evaluation)	Useful context on commissioning design
Physician groups as risk-bearing orgs (delegated model)	USA (California)	Solvency rules for delegated risk & sub-delegation under Title 28.	[78]	State regulation text	Detailed compliance structure for capitation
IDN primary care reform (Geisinger)	USA	ProvenHealth Navigator (PCMH) produced sustained cost reductions over time.	[24]	Longitudinal claims analysis	Early, rigorous PCMH evidence in an IDN
Home-based acute care (Presbyterian)	USA	Hospital-at-Home: 19% lower costs; equal/better outcomes vs. inpatient care.	[28]	Comparative observational study	Frequently cited; programmatic exemplar
Non-profit HMO exemplar (Clalit)	Israel	Nationwide non-profit plans integrating hospitals + primary care under capitation; strong population outcomes.	[29]	System review (HiT)	Authoritative country profile
Capitated PPP exemplar (Alzira)	Spain (Valencia)	Capitated concession integrated primary + hospital care; later reversed via 2018 law; important lessons.	[30]	Policy analysis/case review	Regulatory and political dynamics documented
Vertical integration & prices	USA (commercial market)	Physician–hospital financial integration associated with higher commercial outpatient prices and spending.	[79]	National longitudinal analysis	Found price increases linked to integration
Vertical integration & prices (multi-state)	USA	Vertical integration + joint contracting increased physician prices 2.1–12.0%.	[39]	Econometric analysis (2013–2017)	Quantifies price impact; open access version available
Vertical integration & referral steering	USA	Integration increased referrals to higher-priced facilities (~10%).	[40]	Econometric analysis of referral patterns	Shows the mechanism for spending growth
Facility fees & revenue shifts after integration	USA	Integration boosted facility fees per physician (~$28–$34 k annually), offsetting pro revenue declines.	[41]	Difference-in-differences	Illustrates price-based revenue effects

**Table A4:** Global examples of primary-care-first Integrated Care relevant to India.

Organisation/ Model	Geography	Integration Mechanism	Distinctive Features	Outcomes/ Evidence	Relevance to India
CareMore Health	USA (California; later multi-state)	Physician-led group with its own Medicare Advantage plan	High-risk patient focus; Care centres; extensivists; capitation financing	Reduced hospital admissions and readmissions; improved chronic disease outcomes [80]	Demonstrates how a primary-care-first insurer–provider can scale; urban Indian insurers could replicate for chronic disease management
Physician-led ACOs	USA (Medicare Shared Savings Program)	Independent physician groups leading Accountable Care Organisations	Emphasis on care coordination, outpatient management, chronic disease registries	Consistently greater net savings than hospital-led ACOs [42,75].	Evidence that physician-first integration is financially sustainable under risk contracts
California Delegated Risk Model (IPAs, Medical Groups)	USA (California)	Physician groups (Risk-Bearing Organisations) accept capitation from health plans; subcontract hospital care	Regulatory framework ensures solvency; groups manage utilisation, networks, and referrals	Stable delegated model since 1990s; >50% of Californians in such arrangements [49].	Demonstrates feasibility of physician groups purchasing hospital care—a blueprint for reverse integration in India
GP Fundholding/CCGs	UK (NHS, 1990s–2010s)	General practitioners allocated budgets for defined populations; commissioned hospital/specialist services	Local budgetary authority; referral management; prescribing control	Mixed outcomes; efficiency gains in prescribing, uneven impact on system outcomes [76,81,82]	Shows how government policy can empower primary care with commissioning authority—relevant for PM-JAY/HWC contracting
Maccabi Health Services (contrast to Clalit’s hospital-first)	Israel	Non-profit insurer anchored in strong community-based primary care clinics.	Integrated IT, population health management, specialist contracting.	High satisfaction, chronic disease control, and efficient referral pathways [29]	Illustrates non-profit primary-care-anchored integration under capitation financing

**Table A5:** Comparative pathways to Managed Care Integration in India.

Dimension	Hospital-First Vertical Integration	Primary-Care-First Reverse Integration
Strategic thesis	Use existing hospitals as anchors; add primary care and a financing arm to capture annuity revenues and coordinate utilisation.	Build Starfield-consistent primary care first; integrate upstream by contracting secondary/tertiary services and, where viable, pooling risk.
Best-fit market conditions	Urban/peri-urban; medium–high hospital density; higher competition; payer maturity moderate; capital available.	Rural/remote or mixed settings; low hospital density; payer maturity low; trust deficits; budget-constrained environments.
Starting assets	Beds, specialists, diagnostics, local brand; relationships with employers/insurers.	Longitudinal GP panels, care continuity, community trust, and basic population health data.
Core capabilities required	Network design; plan operations/actuarial; referral management; pharmacy/lab verticals; revenue-cycle and contracting.	Empanelment; risk stratification; nurse-led care management; contracting/reverse-bidding for tertiary care; light TPA/claims functions.
Ownership/financing patterns	Non-profit or for-profit system; may acquire or build a licenced health plan; PE suitable in high-growth corridors.	Non-profit/co-op or hybrid (trust hospital + for-profit insurance subsidiary); donor/blended finance; staged risk delegation with payers.
Capital intensity	High upfront CapEx (clinics, IT, plan capitalisation); Opex for care mgmt.	Low–moderate CapEx (primary care hubs, IT); Opex for care management and contracting; asset-light for tertiary via purchase.
Dominant payment instruments	Capitation (plan-owned lives), risk-adjusted PMPM, bundled payments; selective FFS for out-of-network.	Capitation/delegated risk for defined populations; bundled rates for secondary; tendered tertiary packages via reverse-bidding.
Patient experience	One-brand continuum; faster specialty access; risk of over-medicalisation if incentives poorly governed.	First-contact access, continuity, chronic disease support; clear escalation rules; greater emphasis on prevention and home/virtual care.
Cost/quality evidence (directional)	Programmatic wins (PCMH, hospital-at-home); system-level integration can raise prices without quality gains if unchecked.	Physician-led models show larger savings vs. hospital-led in ACO-style settings; commissioning/delegated risk improves referral/prescribing efficiency.
Key risks	Price inflation, antitrust concerns, weak gatekeeping, capex overreach.	Under-capitalised primary care, weak contracting power, leakage to hospitals, and uneven tertiary quality.
Risk mitigations	Strong utilisation governance, transparent outcomes, phased plan growth, and formulary/diagnostic stewardship.	Contracting playbooks, quality accreditation for referral hospitals, patient navigation, and staged assumption of financial risk.
Global exemplars	Geisinger; Clalit; Alzira (PPP); Unimed (hospital-anchored regions).	California delegated model (RBOs/IPAs); UK GP commissioning; physician-led ACOs.
India feasibility (state archetypes)	Tamil Nadu/Gujarat/Delhi-NCR (dense hospital markets); Kerala/Maharashtra (competitive metros).	Chhattisgarh/Jharkhand/tribal belts (low hospital density); UP/Bihar peri-urban districts (hybrid PC-first + selective hospital upgrades).
Early success metrics (Yr 1–2)	% empanelled lives; ED visits/1000; referral-to-protocol adherence; PMPM trend vs. baseline.	Panel empanelment rate; risk tier coverage; HbA1c/BP control; avoidable admissions; NPS/retention.
Medium-term outcomes (Yr 3–5)	Total cost of care ↓; ACSC (Ambulatory Care Sensitive Conditions) admissions ↓; LOS (Length of Stay) and re-admits ↓; plan MLR (Medical Loss Ratio)	Total cost of care ↓; ACSC admissions ↓; specialist referrals appropriateness ↑; contracted tertiary costs ↓ vs. benchmarks.

**Table A6:** Challenges and opportunities for Indian incumbents.

Incumbent Type	Challenges	Opportunities	Strategy
Internet-based platforms (Practo, 1MG)	Currently function as transaction aggregators with limited clinical accountability; weak integration with offline providers.	Act as digital ‘front doors’ for integrated plans, bundle virtual primary care and navigation, and use data for risk scoring. Opportunity to use the Digital Twin [83,84,85] to shift the momentum of healthcare provision from transactions to relationships.	Partner with insurers to offer subscription/capitation packages; invest in longitudinal outcomes reporting by creating Digital Twins for each patient; build referral and care-coordination capabilities.
Small Family-Owned Hospitals (Maple, Gujarat; Yashoda, Telangana)	Under existential threat from corporate chains and government reimbursement ceilings, thin managerial capacity, and fear of losing fee-for-service revenue streams.	Position as contracted “nodes” within larger integrated networks, diversify into chronic care, and secure annuity-style contracts. Strong local brands and understanding of market dynamics. Doctors as employees/co-owners and not as consultants working on a fee-for-service basis.	Form local alliances or hospital consortia to negotiate as a bloc; partner with digital platforms for patient flows; invest in cost accounting to demonstrate value to integrated buyers. Benefit from the strong local brand to move upstream into primary care and launch an integrated healthcare play with an insurer—work on an arms-length bidding basis with tertiary care providers.
Primary care players (Dvara Health)	Thin margins and low willingness to pay; lack of actuarial skills; nervousness about confronting tertiary specialists & managing downstream “prima donna” doctors.	Become gatekeepers in managed care; capture prevention and chronic care savings; build trust with insurers and employers.	Offer population health contracts; expand through digital monitoring and team-based care; accept risk-adjusted payments with insurer backing [80].
Large Tertiary Systems (Apollo, Narayana)	Dependence on high-margin episodic procedures; strong cultural reliance on consultant free-agents rather than employed doctors, undermining alignment around outcomes.	Anchor integrated managed-care organisations; develop vertically integrated plans; secure annuity revenues through premiums.	Expand into full-service managed health plans; invest in actuarial and population health capabilities; selectively employ specialists to reduce reliance on free-agent consultants; seek an insurance licence.
